# Single and multi-laboratory validation of a droplet digital PCR method

**DOI:** 10.1016/j.foodcont.2022.109117

**Published:** 2022-10

**Authors:** Francesco Gatto, Christian Savini, Maria Grazia Sacco, Daniela Vinciguerra, Gerhard Buttinger, Philippe Corbisier, Marco Mazzara, Hendrik Emons

**Affiliations:** a European Commission, Joint Research Centre (JRC), Via Enrico Fermi 2749, 21027, Ispra, (VA), Italy; b European Commission, Joint Research Centre (JRC), Retieseweg 111, 2440, Geel, Belgium

**Keywords:** Digital PCR, Validation, Trueness, Precision, Genetically modified organisms, Quantification

## Abstract

The authorisation of genetically modified food and feed in the EU is subject to the provision of evidence of safety and of the availability of reliable analytical methods. These methods represent an essential tool for official laboratories to enforce a harmonised market control.

Here the validation of droplet digital PCR (dPCR) methods has been performed for studying if the performance and acceptance parameters set by EU and other international guidelines for the analysis of genetically modified organisms (GMO) in food and feed are suitable and achievable also with such methods. The single-laboratory validation study showed that performance requirements set for GMO analysis by real time PCR can also be used to assess dPCR-based methods. Moreover, trueness and precision were assessed for both simplex and duplex formats in a multi-laboratory validation study organised according to international standards. Overall, the data on trueness, repeatability and reproducibility precision resulting from the collaborative study are satisfying the acceptance criteria for the respective parameters as stipulated in the EU and other international guidance such as the Codex Committee on Methods of Analysis and Sampling (CCMAS). For instance, the duplex droplet dPCR method for MON810 showed relative repeatability standard deviations from 1.8% to 15.7%, while the relative reproducibility standard deviation was found to be between 2.1% and 16.5% over the dynamic range studied. Moreover, the relative bias of the dPCR methods was well below 25% across the entire dynamic range.

In addition, other aspects supporting the application of digital PCR for the control of GMOs on the market were experimentally assessed such as the conversion of the measurement results from copy number ratio to mass fraction, the influence of the DNA extraction step and of the ingredient content. It was found that the DNA extraction step added only a limited contribution to the variability of the measurement results under the studied conditions. The decreasing amount of the target ingredient content may decrease the level of precision of the method, although within the acceptance range of GMO performance parameters.

## Highlights

• In-house and collaborative validation studies of a droplet digital PCR method.
• Trueness and precision assessed and compared for simplex and duplex formats in a collaborative study.
• Evaluation of the influence of DNA extraction and ingredient content.
• Description of compliance to EU and other international performance requirements for GMO analysis methods.
• Application of conversion of measurement results from copy number ratios to mass fractions.

## 1 Introduction

GMO used for food and feed purposes shall be authorised and traceable along the supply chain to comply with legal EU provisions (European Parliament and Council, 2003; European Parliament & Council of the European Union, 2003). Indeed, GMO developers have to provide evidence that their products are safe, together with the availability of suitable tools for official controls. Certified reference materials (CRM) and analytical methods allowing the appropriate detection, identification and quantification of the GMO represent these tools.

To be declared fit for purpose, analytical methods have to fulfil strict performance parameters jointly defined by the European Union Reference Laboratory for Genetically Modified Food and Feed (EURL GMFF) and the European Network of GMO Laboratories (ENGL). The performance parameters are defined in the document “Definition of minimum performance requirements for analytical methods of GMO testing” (ENGL, 2015), which provides guidance on the key performance requirements to be assessed in single- and multi-laboratory validation studies. The requirements laid down in that document are fully in line with globally accepted guidelines (CCMAS, 2010; Horwitz, 1995; ISO, 1994). Until now, a significant number of real time polymerase chain reaction-based methods have been submitted and were successfully validated.

In recent years, digital PCR (dPCR) has emerged as a measurement principle allowing the accurate quantification of nucleic acid targets in various fields, including GMO quantification (Pecoraro et al., 2019). dPCR relies on partitioning the reaction mixture in chambers or water-in-oil droplets of equivalent volume at the nano- or pico-scale, in which the analyte molecules (the target nucleic acid sequence) are randomly distributed and then amplified. The outcome at end-point amplification displays partitions in which the target was either amplified and scored as positive, through the fluorescence emission of a sequence-specific hydrolysed probe, or not amplified and scored negative. Statistics based on Poisson distribution allows estimating the number of copies of the analytical target from the fraction of the partitions scored as positive (ISO, 2019a; Quan et al., 2018).

Compared to real time PCR, dPCR offers the advantage of allowing quantification without the need for external calibration samples, thus reducing the number of reactions and the variability in the experimental set up. Moreover, dPCR is renowned to be less sensitive to PCR inhibitors and more suitable for multiplexing (Demeke and Dobnik, 2018, Whale et al., 2020).

However, dPCR may also present limitations compared to real time PCR. In general, a smaller dynamic range should be expected and is limited by the number of partitions. Unlike in real time PCR, the entire volume of sample loaded into the dPCR is not analysed. It should be considered that a certain amount of sample is not compartmentalised or not accepted for the analysis, constituting a dead volume of reaction. The portion of sample effectively analysed represents a form of subsampling which may lead to an additional uncertainty, especially in the analysis of samples with low amounts of target (Lievens et al., 2016; Pecoraro et al., 2019).

dPCR systems are often based on the use of proprietary reagents, limiting the transferability of methods to other instruments. As of today, dPCR systems present higher costs and hands-on time for the reaction set-up. However, new instruments are coming to the market with competitive performance in comparison to real time PCR.

Single-laboratory validation studies of dPCR methods for GMO analysis have been documented (Demeke et al., 2022; Gerdes et al., 2016; Köppel & Bucher, 2015; Long et al., 2022; Morisset et al., 2013; Noma et al., 2022) and, in some cases, the transferability to a different laboratory was successfully fulfilled (Dobnik et al., 2015; Košir et al., 2017). However, EU legal provisions require to assess the accuracy of a quantitative method in a multi-laboratory validation study conducted in accordance to international standards involving at least 8 laboratories (ENGL, 2015; Horwitz, 1995). Such an extensive assessment of a dPCR method has not been described until now and it thus remained to be verified whether methods based on dPCR could be validated through an international multi-laboratory study.

Another aspect of the application of dPCR-based methods for regulatory purposes regards the expression of results obtained as a copy number ratio of the transgene in relation to a reference gene into the GMO mass fraction (Corbisier et al., 2017). In this context, the use of harmonised conversion factors (CF) was recently proposed (Corbisier & Emons, 2019). The CF is a ratio specifically determined for each GMO CRM enabling the conversion of the relative GM content obtained from measured DNA copies into the corresponding mass fraction. The determination of CFs for each GMO CRM officially accepted for the enforcement of corresponding EU legislation has been recently completed for the currently available CRMs and is continued by the EURL GMFF for newly approved GMOs and new batches of existing matrix GMOs (Corbisier et al., 2022). The application of those CFs allows the harmonised conversion of results originally expressed in DNA copy number ratios - such as those resulting from dPCR measurement data - into GM mass fractions.

The aim of this study was to verify whether the performance of a dPCR method could fulfil the requirements set by EU and other international guidelines (CCMAS, 2010; ENGL, 2015; Horwitz, 1995), including accuracy metrics, when assessed in a collaborative validation study (ISO, 1994; 2019b, 2020). The collaborative study aimed at estimating the trueness and precision by means of repeated measurements on test samples within a concentration range under both repeatability and reproducibility conditions. In this context, it is noteworthy that the same method with identical test items and the same reagents are assayed in order to minimise influences due to components for each factor.

Here a validated real time PCR method for the detection and quantification of MON810 (Mazzara et al., 2009) has been assessed. The method was already adapted to dPCR and assessed in a single-laboratory study either in simplex and duplex formats (Morisset et al., 2013).

In addition, a matrix CRM together with samples containing decreasing amounts of DNA were distributed to participating laboratories for evaluating the influence to the accuracy metrics of the DNA extraction procedure and of the amount of target.

## 2 Materials and methods

### 2.1 Test materials

CRMs of the GM event MON810 were obtained from the European Commission's Joint Research Centre (JRC). In particular, the following CRMs were used: ERM-BF413ck (4.9 g/kg, 0.49% m/m), ERM-BF413ek (19.8 g/kg, 1.98% m/m), ERM-BF413gk (99 g/kg, 9.9% m/m). The ERM-BF413ak (blank, < 0.9 g/kg) is containing traces of the target GM event MON810 (Broothaerts et al., 2009). Therefore, seeds of non-modified maize were purchased from a local retailer and tested for the absence of contamination by MON810.

### 2.2 DNA extraction

Genomic DNA was extracted from CRM powders using the Foodproof sample preparation kit III (Biotecon Diagnostics, Potsdam, Germany) and then quantified by using the Quant-iT PicoGreen dsDNA Assay Kit (Molecular Probes, Eugene, USA). The quality of each of the DNA extracts was checked and accepted, if it complied with the minimum acceptance criteria for DNA extraction (ENGL, 2015). DNA samples were stored at 4 °C until further use.

For each DNA sample, the absence of PCR inhibitors was assessed through a real time PCR inhibition run, carried out as described in Annex 2 in Hougs et al., 2017.

### 2.3 Sample preparation

GM mass fractions of 4.9, 19.8 and 99 g/kg of MON810 were extracted from the corresponding CRMs. Samples containing 1.0 and 9.0 g/kg of MON810 were obtained by blending the DNA solutions from non-modified maize with those obtained from the CRMs with 4.9 and 19.8 g/kg of MON810, respectively. Mixtures were prepared considering the concentration in copies of the reference gene *high mobility group* (*hmg*) determined by droplet digital PCR. The uncertainty of the GM content of these materials was determined considering the uncertainty contributions from the purity of the CRM, the determination of the DNA concentration and the pipetting. The detailed procedure is described in the Section A of the Electronic Supplementary Material (ESM).

Mass fractions and DNA copies per reaction of each target are displayed in Table 1. The theoretical values for the number of copies of *hmg* and MON 810 targets have been estimated assuming a value of 2.73 pg for the haploid genome size (1C) (Bennett & Leitch, 2012) and a ratio of 0.42 for MON810/hmg considering the male donor parent for the GM event (Zhang et al., 2008). These values and the theoretical estimates of the copies per reaction are used as reference values throughout the assessment of the validation parameters.

**Table 1:** Estimated total DNA mass partitioned across the droplets and theoretically estimated number of copies of *hmg* and MON810 targets per reaction (cp/rxn) under the assumptions described in the text above; all values are presented without uncertainties. 1:10 and 1:100 indicates the dilution ratio of the sample.

Certified value (g/kg)	1.0	4.9	9.0	19.8 (0.77%^3^)	99	99_1:10_	99_1:100_
Uncertainty	0.2^1^	1.0^2^	0.7^1^	1.5^2^ (0.08%^4^)	5^2^	5^2^	5^2^
DNA amount (ng)	200	200	200	200	200	20	2
*hmg* (cp/rxn)	73260	73260	73260	73260	73260	7326	733
MON810 (cp/rxn)	31	151	277	609 (564^5^)	3046	305	30

^1-2^ Expanded uncertainty expressed with the confidence of 95% (*k* = 2) estimated from the preparation^1^or from the CRM provider^2^.

^3-4^ Certified value in copy number ratio^3^ and expanded uncertainnty^4^ with the confidence of 95% (*k* = 2).

^5^ MON810 copies per reaction using as reference the certified value in copy number ratio.

### 2.4 Droplet digital PCR set up

Primers and probes sequences were those of the validated real time PCR method (Mazzara et al., 2009). The probe targeting MON810 was labelled with FAM, while the probe for the *hmg* was labelled with HEX.

Droplet digital PCR solutions were prepared for a final reaction volume of 20 μL either for simplex or duplex reaction set ups. The compositions of the reaction mixtures were: 1x ddPCR Supermix for probes (No dUTP) (BioRad, Hercules, USA), 450 nM of each primers, 200 nM of probes. Droplets were generated by using QXDx AutoDG Droplet Digital PCR Systems (BioRad). PCR runs were carried out by using a C1000 Touch Thermal Cycler (BioRad) under the following amplification conditions: 10 min at 95 °C; 40 cycles of 30 s at 94 °C and 60 s at 60 °C; followed by 10 min at 98 °C; and then cooled down to 4 °C. The ramp rate was set at 2.0 °C/s. After thermal cycling, the 96-well plates were transferred to the droplet reader QX200 (BioRad) and data were gathered and analysed using the BioRad QuantaSoft software (Version 1.7.4.0917).

### 2.5 Determination of the GM content

The relative MON810 content expressed as copy number ratio (% cp/cp) was calculated from the ratio between the copies of MON810 and the maize endogenous gene *hmg*.

The relative content of MON810 is then expressed in mass fractions (g/kg) by dividing the copy number ratio by the conversion factor (CF) of 0.36 ± 0.04 (k = 2) as determined by the EURL GMFF (Conversion factors (CF) for CRM, estimates on ERM®-BF413gk, https://gmo-crl.jrc.ec.europa.eu/doc/CF-CRM-values-v5.pdf) (EURL GMFF, 2019) and then multiplied by 1000 to obtain the result in g/kg:

$$w_{MON810}=\frac{C_{MON810}}{C_{hmg}}\times \frac{1000}{CF_{MON810}}$$

where:

*w*_MON810_ is the mass fraction of MON810 in the sample, expressed in g/kg.

*C*_MON810_ is the number of copies of the MON810 target per reaction.

*C*_*hmg*_ is the number of copies of the *hmg* target per reaction.

*CF*_MON810_ is the conversion factor for the certified value of CRM ERM®-BF413gk.

The number of target copies per reaction was estimated by applying the formula below derived from Formula 3 in ISO 20395 (ISO, 2019a), taking into account a reaction volume of 20 μL and an average volume for a partition of 0.786 nL (Emslie et al., 2019):

$$C_{cp/rxn}=20\;\mathrm{\mu L\times}\left[-\ln\left(1-\frac{N_{p}}{N_{T}}\right)\right]\text{×}\frac{10^{3}}{0.786\;\text{nL}}$$

where:

*C*_cp/rxn_ is the number of target copies per reaction.

N_p_ is the number of positive partitions.

N_T_ is the total number of accepted partitions.

### 2.6 In-house validation

The in-house validation of the duplex dPCR MON810 method was performed in order to evaluate trueness and precision within the dynamic range under repeatability conditions, the limit of quantification (LOQ), the limit of detection (LOD), the robustness and linearity.

Trueness and precision were assessed in terms of relative bias and relative repeatability standard deviation (RSD_r_), respectively, from results obtained by analysing four sample replicates in three runs each, resulting in a total of 12 data points per GM level. From the same data, the dynamic range, the LOQ and linearity were determined.

The linearity was evaluated as the mean of the slope and R^2^ from the linear regression obtained on a plot of measured against reference values according to ISO 20395 (ISO, 2019a).

The LOD was evaluated on the basis of the analysis of 64 replicate measurements of one sample containing 0.45 g/kg (0.045% m/m) of MON 810.

The robustness of the method was verified by introducing small deviations from the experimental conditions of the protocol. Here, a multifactorial study design was applied in order to assess the following modifications: 1) temperature ramp rate (unchanged/+ 0.5 °C); 2) annealing temperature (unchanged/+ 1 °C); 3) primer and probe concentrations (unchanged/- 10%); 4) mastermix concentration (unchanged/- 10%); The RSD_r_ and trueness were determined by the analysis of a sample with a GM content close to the LOQ (*i.e.* 1 g/kg).

### 2.7 International collaborative trial

The collaborative trial was organized by the EURL GMFF in December 2020 and January 2021: 14 laboratories, all members of the ENGL network, took part in the study based on the experience with droplet digital PCR (see list of participating laboratories in ESM). The droplet dPCR method was assessed both in simplex and duplex configurations with the aim to evaluate and compare the performance parameters.

Each laboratory received a kit for the study that included all reagents, samples and disposable plastic materials needed, with the exception of the deionised water. Samples and reagents were shipped to the laboratories under temperature-controlled conditions (+4 °C), while the disposable plastic materials were shipped at room temperature.

A detailed protocol and an Excel file for reporting results were distributed together with a link to a questionnaire for the documentation of the instruments used, protocol deviations and information about the sample preparation for the analysis.

Test samples covered a range of GM mass fractions centred at 9.0 g/kg (0.9% m/m), i.e. the labelling threshold in the EU, and spanned from 99 down to 1.0 g/kg of GM material. The latter lowest content level corresponds to the minimum mass fraction of GMO that should be quantifiable with an acceptable level of trueness and precision (European Commission, 2011).

The extract from the sample with 99 g/kg GM was also measured in various dilutions: undiluted or diluted 1:10 and 1:100 with the buffer 0.1x TE (Tris, EDTA, pH 8.0) simulating samples with a decreasing content of total maize DNA (these samples are indicated through the text as 99_1:10_ and 99_1:100_, respectively). This GM level was selected in order to have a suitable number of target copies of the GM event and of the taxon present for quantification (see Table 1).

Moreover, one sample was distributed both as DNA solution and as powder CRM to be extracted in four replicates, similarly to the same sample distributed as DNA solution, to evaluate a potential influence of the DNA extraction step. Participating laboratories extracted genomic DNA by using a method of their choice. A CTAB-based approach was followed most frequently (CTAB: n = 6, CTAB-Maxwell: n = 3), in other cases silica-membrane-based approaches were followed (Machery-Nagel Nucleospin Food kit: n = 3, or other: n = 2).

Each laboratory performed two runs according to the instructions provided, analysing in parallel the same samples by simplex and duplex reaction set ups. Each of the four sample replicates was analysed with two PCR reactions. A no-template control was included on the plate.

Two measures of accuracy have been evaluated in accordance with ISO 5725: trueness and precision. Trueness was expressed as relative mean bias (difference between the mean value of the test results and the accepted reference value divided by the measured GM content). Precision was expressed as relative repeatability standard deviation (RSD_r_) and as relative reproducibility standard deviation (RSD_R_) with respect to the measured GM content, respectively.

The identified outlying data and/or laboratories were excluded using Cochran and Grubbs tests according to ISO 5725 (ISO, 2019c).

Consistency between measured versus reference values was assessed by comparing their difference to the measurement uncertainty estimated with a level of confidence of 95%.

The measurement uncertainty was calculated in two steps. First the standard uncertainty for the measurement and for the bias have been estimated separately according to the Guidance for measurement uncertainty for GMO analysis (Trapman et al., 2020) and then combined:

$$u=\sqrt{u_{m}^{2}+u_{bias}^{2}}$$

where.

*u*_*m*_ is the uncertainty of the mean results (measured under repeatability conditions);

*u*_*bias*_ is the uncertainty related to the bias (from CRM measurements).

Afterwards, the uncertainty of the applied CF (*i.e.* 0.04 (k = 2)) was included following the approach described in the corresponding Application Note of the EURL GMFF (EURL GMFF, 2019).

## 3 Results and discussions

### 3.1 In-house validation

The single-laboratory study was performed for the duplex dPCR MON810 method to assess trueness and precision within the dynamic range, the limit of quantification (LOQ), the limit of detection (LOD), robustness and linearity.

This step was carried out in a way which would be expected to be performed by an applicant under Reg. (EC) No 1829/2003 (European Parliament and Council, 2003) following the ENGL guidance document to demonstrate that defined method performance requirements are met. Although performance requirements tailored for the peculiarities of dPCR methods are still under elaboration and have not been published at the time of designing this study, the applicable metrics for real time PCR methods (ENGL, 2015) currently in force have been considered together with parameters which could be derived from other published literature, ENGL recommendations (Pecoraro et al., 2019) and an ISO standard (ISO, 2019a).

Specificity was not experimentally assessed, however an *in silico* analysis was carried out confirming the theoretical specificity of the method (data not shown). Amplification efficiency and R^2^ are quality parameters for a calibration curve and therefore not applicable to the dPCR set up.

#### 3.1.1 Dynamic range, Limit of quantification (LOQ), trueness and precision

Trueness and precision are two key performance parameters, as they are describing the accuracy of results of a quantitative method. Given a set of measurement results, the trueness indicates the degree of agreement of their mean to the reference (true) value, while precision is the closeness among the set of results. The range of concentrations of the analyte over which the method showed a suitable level of trueness and precision is here defined as dynamic range. In GMO analysis, a relative bias and a relative repeatability standard deviation (RSD_r_) of 25% are considered suitable for trueness and precision, respectively (CCMAS, 2010; ENGL, 2015).

In this study the accuracy was assessed measuring the relative content, in mass fraction (g/kg), and the copies per reaction of MON810.

Indeed, the results in mass fraction in Table 2 showed a maximum bias of 7.4% for measurements at 4.9 g/kg and a maximum RSD_r_ of 15.7% at the lowest concentration level.

**Table 2:** Trueness (relative bias) and precision (RSD_r_) obtained during the in-house testing within the dynamic range (calculated in g/kg and copies per reaction (cp/rxn), respectively).

	GM level in g/kg				GM level in copies/reaction			
Sample	GM level (g/kg)	Measured mean (g/kg)	Bias (%)	RSD_r_ (%)	GM level (cp/rxn)	Measured mean (cp/rxn)	Bias (%)	RSD_r_ (%)
A	1.0	1.1	6.0	15.7	31	31	0.3	18.9
B	4.9	5.3	7.4	7.4	151	143	−5.3	8.7
C	9.0	9.5	5.9	4.7	277	258	−6.9	9.1
D	19.8	20	3.3	4.5	609	569	−6.6	6.3
E	99	100	1.1	1.6	3046	2900	−4.8	3.6
F	99_1:10_	99	−0.1	5.2	305	284	−6.8	7.9
G	99_1:100_	102	2.9	12.3	30	31	1.1	12.0

Similarly, a maximum bias of - 6.9% was observed for the sample with 277 copies/reaction and a maximum RSD_r_ of 18.9% for the measurement of the sample with 30 copies/reaction (Table 2). Even if results do not show major differences, it should be noted that the calculation of the target copies per reaction required a different approach with respect to the ratio. For instance, the average volume of the partition (i.e. 0.786 nL according to Emslie et al., 2019), which is not relevant for the GM ratio calculation, has to be considered.

Therefore, the duplex dPCR method fulfils the requirements for trueness and precision according to the acceptance criteria defined by the ENGL. Thus, the dynamic range is covering at least GM mass fractions from 1.0 to 99 g/kg and target DNA amounts from 30 to 3046 copies/reaction of MON 810, respectively.

Interestingly, the sample with a 19.8 g/kg MON810 content is certified also for its content in terms of copy number ratio (i.e. 0.77% ± 0.08%, k = 2). The analysis of this sample allows to compare measurements in terms of copies obtained by two independent measurement principles. In fact, the certified value was obtained by real time PCR and calibrated by a plasmid carrying a single copy of MON810 and *hmg* targets (Broothaerts et al., 2009). The mean ratio measured by dPCR was 0.74%, which is within the certified range taking uncertainties into account, and is confirming the agreement between measurement results obtained by the two techniques. The good accordance of values is also confirmed, when the absolute copies per reaction of MON810 are estimated by using the certified copy number ratio instead of the theoretical assumptions of the expected zygosity and GM content. Indeed, 564 copies per reaction of MON 810 are expected for 200 ng of DNA input, which it is close to the measured of 569 copies per reaction (Table 2).

From the same data, the LOQ, *i.e.* the lowest concentration of analyte that can be quantified with an acceptable level of trueness and precision, was assessed on the same sample and found as equal or below 1.0 g/kg (0.1% m/m) or 31 copies/reaction. This is confirming the accuracy of the method when measuring small amounts of GM target.

#### 3.1.2 Linearity

**Fig. 1:**
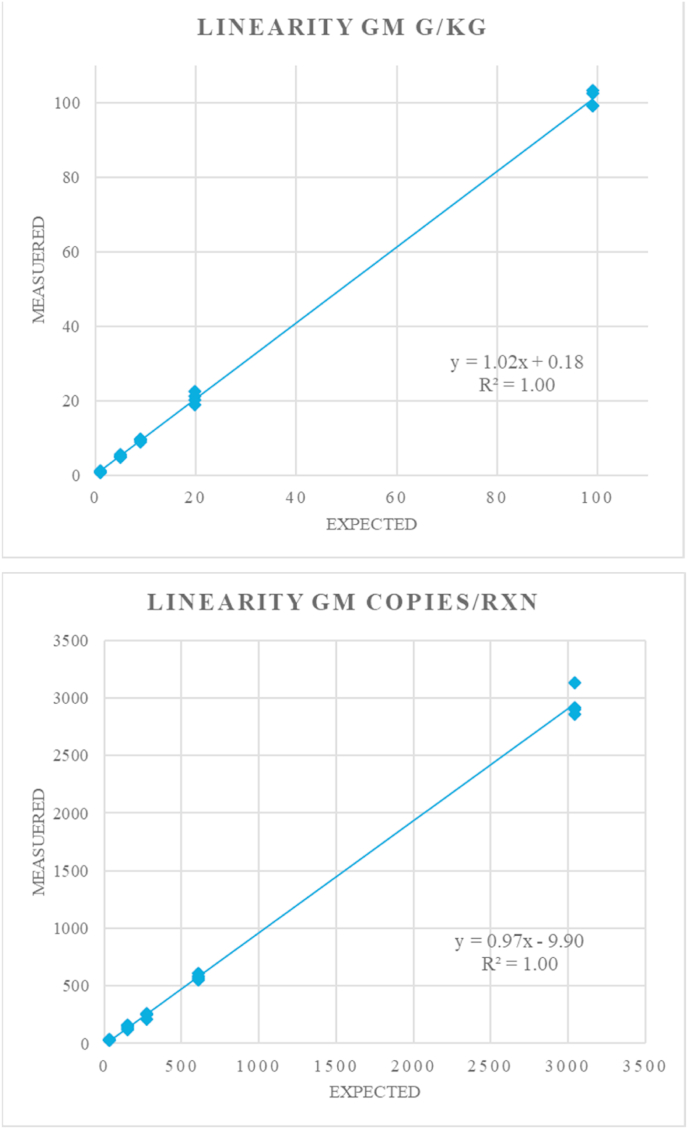
Example for the linearity of dPCR results. Top: Relative content of MON 810 expressed in g/kg; Below: MON 810 content expressed in copies per reaction.

The linearity is the ability of a method of analysis to provide within a specified range an instrumental response or results directly proportional to the content of the analyte in the test sample. The same data from the analysis of the dynamic range were also analysed for the evaluation of linearity. The degree of linearity was assessed by the slope and the R^2^ of the regression line of the observed vs. expected target amounts for the GM content in mass fraction (Fig. 1A) and in GM copies per reaction (Fig. 1B) observed for each of the three runs. A recent international standard for quantitative methods for nucleic acid targets (ISO, 2019a) is recommending a value of the slope of 1.00 ± 0.05 and of the R^2^ greater than 0.99.

The average slope of a plot of “measured vs. expected values” of the copies of the targets (i.e. MON810 cp/rxn) or the GM mass fractions (i.e. g/kg) was found to be within the recommended range for the relative quantification, with a slightly larger deviation for the results of one experimental run (Table 3). The coefficient of determination (R^2^) was always 0.99 or higher, indicating a good fit of the data with the linear model.

**Table 3:** Outcome of the linear regression between measured GM content and expected values expressed in different measurement units.

	Linearity GM Level in g/kg			Linearity GM level in copies/reaction		
Run	Slope	intercept	R^2^	Slope	Intercept	R^2^
1	1.02	0.18	1.00	0.94	−3.28	1.00
2	1.00	0.44	1.00	0.97	−9.90	1.00
3	1.01	0.32	1.00	0.95	−3.83	1.00
**Mean**	**1.01**	**0.32**	**1.00**	**0.96**	**−5.67**	**1.00**

#### 3.1.3 Limit of detection (LOD)

The limit of detection is the lowest amount or concentration of an analyte in a sample that can be reliably detected, but not necessarily quantified. It is commonly recognised that analytical methods should allow the detection of an amount of the analyte with a level of confidence of 95%, ensuring less than 5% of false negative results.

A suitable number of replicates are needed to achieve the confidence level. The ENGL recommends to perform at least 60 PCR replicates, of which 59 should indicate at the LOD that the analyte is detected (ENGL, 2015). A sample with 0.45 g/kg (0.045% m/m) of MON810 was tested in line with the above guidance. 63 out of 64 dPCR replicates provided valid results (i.e. >10000 droplets measured) and all of them showed positive results (i.e. > 2 positive droplets) corresponding to the detection of 13 GM copies per reaction (see section B in ESM).

While a real time PCR measurement is providing a single signal during amplification to indicate the presence of the target sequence in the whole reacting sample, thousands of end-of-amplification signals are generated in dPCR as a result of the volumetric partitioning of the reacting sample. In principle, even a single fluorescent partition within a total of thousands could be interpreted as a detection of the target nucleotide sequence. However, practical experience with droplet dPCR showed sporadically that fluorescent partitions could be observed also in negative samples. Therefore, the ENGL has recommended as a practical guidance that up to 2 fluorescent partitions may not be considered as sufficient to prove that the target sequence is detected with a suitable level of confidence (Pecoraro et al., 2019). This approach is taken here as a guide for the interpretation of qualitative results and is in line with the instrument manufacturer recommendations (Biorad, 2018). Tests performed in this study on MON810 and *hmg* targets showed that the LOD is 10 copies/reaction when at least 3 fluorescent droplets are considered for the qualification of positive reactions. Detailed results are displayed in section B in ESM.

#### 3.1.4 Robustness

The assessment of the impact of small deviations from the experimental conditions in a single laboratory study allows the identification of possible issues when the method would be used for a longer time and/or by different operators, but in particular for its transfer to other laboratories. In real time PCR, a number of factors were identified as critical (ENGL, 2015) and had been tested in multi-factorial studies. In this study, a few adaptations to the experimental design were applied and tested in combination. The RSD_r_ and trueness have been calculated for the results obtained by measuring the 1.0 g/kg GM sample. In line with the ENGL guidance, precision and trueness were found to not exceed 30% for a combination of changes (see section B in ESM).

### 3.2 International collaborative trial

The collaborative validation study was aiming to determine method's repeatability, reproducibility and trueness and to assess them against predefined performance requirements (CCMAS, 2010). The latter are those established by the ENGL and adopted by the EURL GMFF (ENGL 2015) for methods for the detection and quantification of GMOs submitted in support of the EU authorisation.

#### 3.2.1 Precision

The relative repeatability standard deviation (RSD_r_) is a measure for the variation expected from results generated in a single laboratory, while the relative reproducibility standard deviation (RSD_R_) is a parameter for the inter-laboratory variation.

Table 4 shows the performance of the methods in terms of precision under repeatability and reproducibility conditions in the simplex and duplex dPCR formats, respectively.

**Table 4:** Performance parameters obtained for the simplex (S) and duplex (D) ddPCR methods (14 laboratories). (D_ext_ refers to the fluor sample analysed by duplex ddPCR after DNA extraction performed by each laboratory)

	MON 810 concentration levels (g/kg)														
	1.0		4.9		9.0		19.8			99		99_1:10_		99_1:100_	
	S	D	S	D	S	D	S	D	D_ext_	S	D	S	D	S	D
Number of outliers and reason for exclusion^a^	–	–	–	–	–	–	1C	–	–	–	–	1C	1 DG	–	–
Measured GM content (g/kg)	1.1	1.0	5.7	5.6	9.4	9.3	20.5	20.9	20.1	101.5	101.0	101.2	101.3	100.6	100.4
Repeatability standard deviation (g/kg)	0.2	0.2	0.5	0.4	0.7	0.5	1.0	0.9	1.4	4.6	1.9	5.6	6.9	14.9	16.3
Relative repeatability standard deviation, RSD_r_ (%)	19.0	15.7	8.9	7.7	7.4	5.7	4.9	4.4	6.8	4.5	1.8	5.6	6.8	14.8	16.3
Reproducibility standard deviation (g/kg)	0.2	0.2	0.5	0.4	0.8	0.6	1.1	1.0	1.5	4.6	2.2	7.0	6.9	16.2	17.4
Relative reproducibility standard deviation, RSD_R_ (%)	19.0	16.5	8.9	7.7	8.1	6.2	5.6	5.0	7.3	4.6	2.1	7.0	6.8	16.1	17.4
Bias (g/kg)	0.1	0.0	0.8	0.7	0.4	0.3	0.7	1.1	0.3	2.5	2.5	2.2	2.2	1.6	1.6
Relative bias (%)	10.7	3.8	17.0	13.5	4.3	3.5	3.6	5.3	1.7	2.5	2.5	2.2	2.2	1.6	1.6
Expanded measurement uncertainty (g/kg, *k* = 2, confidence level 95%)	0.4	0.4	1.6	1.5	1.7	1.5	3.2	3.2	3.4	14.1	12.7	16.2	15.9	26.7	27.8

a C, Cochran test; G, Grubbs tests, DG, Double Grubbs test.

**Fig. 2:**
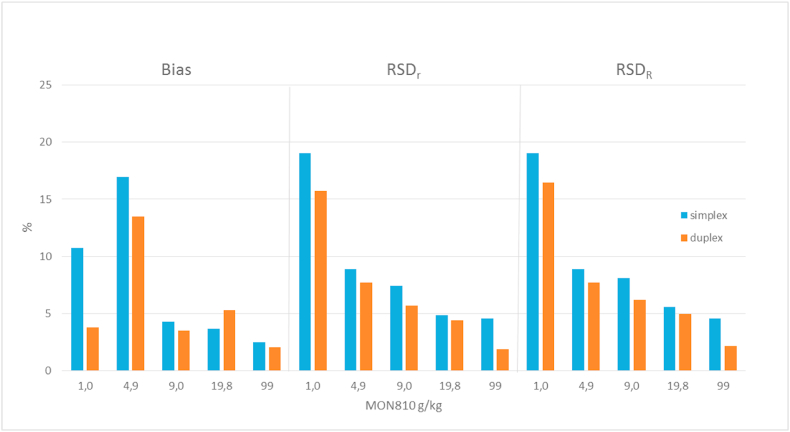
Trueness and precision: relative bias (%), relative repeatability (RSDr) and reproducibility (RSD_R_) standard deviations for simplex (blue) and duplex (orange) versions of the dPCR method.

The duplex droplet dPCR method for MON810 showed RSD_r_ values ranging from 1.8% to 15.7%, while the RSD_R_ was found to be between 2.1% and 16.5% over the dynamic range (Table 4). The simplex dPCR method displayed a slight decrease in precision in comparison to the duplex set up (Fig. 2) resulting in higher values for RSD_r_ (between 4.5% and 19.0%) and RSD_R_ (between 4.6% and 19.0%) (Table 4). However, the difference in the precision metrics between simplex and duplex setups is rather small and could, probably, be caused by the need of more pipetting steps of reagents and templates in the simplex setup.

According to the EU performance requirements (ENGL, 2015) and other international criteria set in the Codex Alimentarius (CCMAS, 2010), the RSD_r_ should be below 25% across the dynamic range and the RSD_R_ should be below 35% and below 50% for a GM content below 0.2% m/m (ENGL, 2015). The dPCR method complied well to these requirements in both simplex and duplex setups.

#### 3.2.2 Trueness

The trueness of a method is assessed in terms of relative bias, which is calculated as the relative difference of the measured and the reference value.

The range of bias for the duplex dPCR method was 2.5%–13.5%, while it varied from 2.5% to 17% for the simplex dPCR method (Table 4).

Overall, the biases of the dPCR methods are well below 25% across the entire dynamic range, thus confirming that the performance criteria established for real time PCR (ENGL, 2015) could also be applicable to digital PCR methods, for both simplex and duplex setups.

Fig. 2 showed that the trueness is not substantially affected by the setup of the method. A difference in terms of bias can be observed at the lowest GM content level tested (1.0 g/kg): 3.8% and 10.7% for duplex and simplex setup, respectively. However, the absolute difference between the two measurement results are not significant when the associated measurement uncertainty is taken into account (i.e. 1.0 ± 0.4 g/kg and 1.1 ± 0.4 g/kg).

The agreement between measured and reference values is also confirmed for the results observed on all samples and obtained by both dPCR setup. In all cases, the absolute bias between the mean and the reference is always smaller than the measurement uncertainty at the level of confidence of 95% (Table 4).

#### 3.2.3 Impact of DNA extraction

One sample was analysed either as DNA solution prepared by the provider and as powder from which each participants had performed the DNA extraction before the dPCR analysis.

Participants selected the method according to their own procedure resulting in 9 laboratories using CTAB-based and 5 laboratories using silica membrane-based methods. No difference was observed on results from the two groups of DNA extraction approaches (data not shown).

**Fig. 3:**
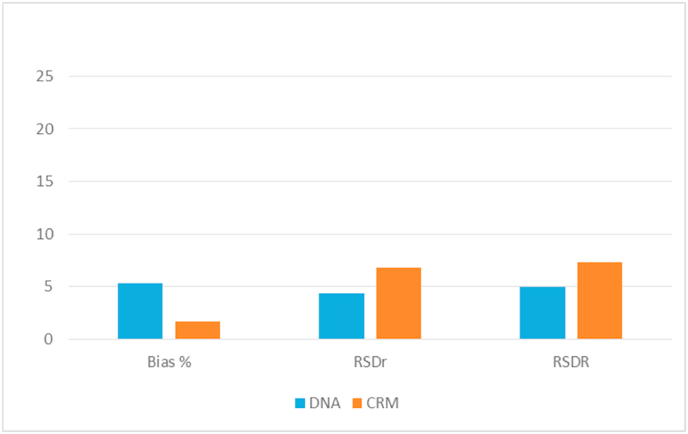
Comparison of trueness and precision on sample at 19.8 g/kg MON 810 content analysed in the collaborative study and distributed by the provider either as DNA solution (blue) and certified powder (CRM, orange).

Fig. 3 shows the comparison of the performance metrics observed from the analysis of the sample with 19.8 g/kg GM. The results are equivalent and no significant difference was observed between the measurement results on DNA from solution vs the one from the powder sample. A small increase of the relative standard deviations was found, resulting in a slightly higher measurement uncertainty (i.e. ± 3.2 g/kg for DNA sample and ±3.4 g/kg for powder sample measurements). However, the differences observed are very small indicating that the DNA extraction step may have a negligible impact, especially when simple matrix materials such as CRMs are analysed.

#### 3.2.4 Influence of ingredient content

It has also been investigated, if the quantification by dPCR would be affected when measuring a food mixture in which the ingredient ‘maize’ corresponds only to a small portion of the constituents of the samples. The test material containing 99 g/kg of MON 810 (about 10% GMO content) was analysed in an undiluted as well as in diluted stages (1:10 and 1:100 with buffer). These diluted samples were containing the same relative MON810 content. However, the number of MON810 target copies was decreased from 3046 to 305 and 30 copies, respectively, in the corresponding diluted samples (Table 1). The latter number of MON810 copies is actually the same as present in an undiluted extract from the 1.0 g/kg GM sample, although with much less total maize genome copies.

**Fig. 4:**
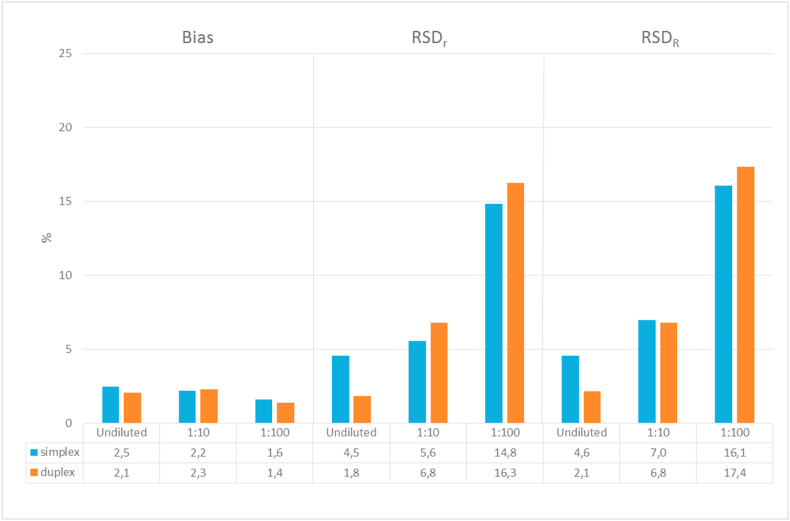
Comparison of trueness and precision of simplex (blue) and duplex (orange) ddPCR measurements of the sample at 99 g/kg MON 810 content analysed in undiluted and diluted levels.

The results displayed in Fig. 4 show that the mean values measured after such dilutions are similar. Indeed, almost identical measured values were observed over the dilution series with both simplex and duplex setups.

As expected, the precision decreased with the dilution. Interestingly, RSD_r_ and RSD_R_ for the measurement results on the 99 g/kg GM sample diluted 1:100 are in the same range as the ones observed for analysing the 1.0 g/kg sample. These data are indicating that a sample with a very low content of the target GM analyte (*i.e.* 30 copies of MON810 in the 1:100 diluted sample) could be correctly measured by a well-performing analytical method, even if its controlled ingredient (e.g. maize) would constitute only a minor part of the whole marketed food sample.

## 4 Conclusions

This study presents the validation of a droplet digital PCR method assessed through both single-laboratory and collaborative validation studies.

The data presented from the single-laboratory validation study showed that performance requirements set for GMO analysis by real time PCR can also be used to assess dPCR-based methods. Some validation parameters, such as those related to the calibration of real time PCR methods, are not applicable to digital PCR methods.

The good agreement of reference and measured GMO mass ratios, observed by trueness and linearity, demonstrates that the use of the conversion factors for digital PCR analysis of GMO is providing a suitable tool for the transformation of measurement units, thus allowing the enforcement of the EU official control legislation. Overall, the data on trueness, repeatability and reproducibility precision resulting from the collaborative study are also well in agreement with the acceptance criteria for the respective parameters as stipulated in the Codex Committee on Methods of Analysis and Sampling (CCMAS).

The method assessed both in simplex and duplex set-ups provided suitable precision under repeatability and reproducibility conditions, respectively. It was found that a slight increase in the variation of measurement results could be expected when the relative GM content is obtained from data originating from simplex compared to duplex dPCR methods. The DNA extraction step seems to add only a limited contribution to the variability of the measurement results when single-ingredient non-heated powder mixtures are analysed. On the other hand, a decreasing amount of the target ingredient content may decrease the level of precision of the method, although both RSD_r_ and RSD_R_ were in our study within the acceptance range of GMO performance parameters according to EU and other international guidelines.

In summary, the study demonstrated that a validation of droplet dPCR methods, including the assessment of the accuracy in collaborative validation studies, can be performed in line with international standards (CCMAS, 2010; ISO, 1994) and in agreement with EU requirements (ENGL, 2015).

## Declaration of competing interest

The authors declare that they have no known competing financial interests or personal relationships that could have appeared to influence the work reported in this paper.

Certain commercial equipment, instruments, and materials are identified in this manuscript to specify adequately the experimental procedure. Such identification does in no case imply a recommendation or endorsement by the European Commission, nor does it imply that the material or equipment is necessarily the best available for the purpose.

## CRediT authorship contribution statement

**Francesco Gatto:** Conceptualization, Methodology, Validation, Formal analysis, Investigation, Writing – original draft, Supervision, Project administration. **Christian Savini:** Conceptualization, Methodology, Writing – review & editing, Supervision. **Maria Grazia Sacco:** Conceptualization, Validation, Investigation, Data curation. **Daniela Vinciguerra:** Conceptualization, Co nceptualization, Methodology, Validation, Investigation. **Gerhard Buttinger:** Conceptualization, Investigation, Writing – review & editing. **Philippe Corbisier:** Conceptualization, Investigation, Writing – review & editing. **Marco Mazzara:** Conceptualization, Project administration, Writing – review & editing. **Hendrik Emons:** Conceptualization, Writing – review & editing.

## Declaration of competing interest

o This manuscript has not been submitted to, nor is under review at, another journal or other publishing venue.

o The authors have no affiliation with any organization with a direct or indirect financial interest in the subject matter discussed in the manuscript.
