# Supplementary material for: Single and multi-laboratory validation of a droplet digital PCR method
Source: Food Control. 2022 Oct;140:109117. doi: 10.1016/j.foodcont.2022.109117 (PMC9231119; doi:10.1016/j.foodcont.2022.109117)
Supplement: Multimedia component 1 [file mmc1.pdf]

Single and multi-laboratory validation of a droplet digital PCR method

Electronic Supplementary Material

Section A: Uncertainty of the reference value

Samples (reference materials) containing 1.0 and 9.0 g/kg of MON810 were prepared by mixing DNA extracted from certified reference materials (CRM) with DNA extracted from non-GM maize (NGM).
The concentrations in terms of copies/µL were determined by droplet digital PCR using the method for the taxon-specific *hmg* target.
The preparation can be described by the following formula (Eq. 1S):

$$w_{\mathrm{RM}} = \frac{c_{\mathrm{CRM}} \times v_{\mathrm{CRM}} \times w_{\mathrm{GM,CRM}}}{c_{\mathrm{NGM}} \times v_{\mathrm{NGM}} + c_{\mathrm{CRM}} \times v_{\mathrm{CRM}}}$$

where:
w_{RM}: GM mass fraction of the prepared reference material (in g/kg)
c_{CRM} : concentration (in copies/µL) of the *hmg* target in the DNA solution extracted from the CRM
v_{CRM} : volume (in µL) taken of the DNA solution extracted from the CRM
$w_{\mathrm{GM,CRM}}$: certified value for the GM mass fraction of the CRM (in g/kg)
c_{NGM} : concentration (in copies/µL) of the *hmg* target in the DNA solution extracted from the NGM
v_{NGM} : volume (in µL) taken of the DNA solution extracted from the NGM

The uncertainty of w_{RM} was estimated following the approach described in Section 7.2.2 in Trapmann et al. (2014)[1] as well as by applying the error propagation principle to the parameters of equation above.
The following uncertainty sources were considered:

- uncertainty of the *hmg* concentration of the DNA solution extracted from the CRM ($u_{\mathrm{c,CRM}}$): estimated as standard error of the mean of c_{CRM};
- uncertainty of the *hmg* concentration of the DNA solution from the non-GM material ($u_{\mathrm{c,NGM}}$): estimated as standard error of the mean of c_{NGM};
- uncertainty associated with the certified value of the CRM used ($u_{\mathrm{w,CRM}}$): standard uncertainty of the certified value taken;
- uncertainty associated with the volume of the taken GM DNA solution ($u_{\mathrm{v,CRM}}$): estimated as standard error of the pipette volume taken from the technical specification of the pipette;
- uncertainty associated with the volume of the taken NGM DNA solution ($u_{\mathrm{v,NGM}}$): estimated as standard error of the pipette volume taken from the technical specification of the pipette.

[1] Trapmann, S., Charles Delobel, C., Corbisier, P., Emons, H., Hougs, L., Philipp, P., Sandberg, M., Schulze, M. (2014) European technical guidance document for the flexible scope accreditation of laboratories quantifying GMOs, Luxembourg, Publications Office of the European Union. https://data.europa.eu/doi/10.2787/90706

Table 1S: Combined standard (u) and expanded (U) uncertainties of the GM mass fractions of the prepared reference materials using two approaches

	**1 g/kg**		**9 g/kg**	
	via Trapmann et al. (2014)	via error propagation	via Trapmann et al. (2014)	via error propagation
$u_{w,RM}$ (g/kg)	0.11	0.11	0.38	0.37
$U_{w,RM}$ (k=2) (g/kg)	0.21	0.21	0.76	0.73

Section B: Results from in-house testing

Limit of detection (LOD)

Detailed results for the evaluation of the LOD from the analysis of a sample containing 0.045 % m/m MON810 in 63-64 replicates

Table 2S: Relative LOD. Results of the analysis of the sample level 0.45 g/kg (13 GM copies/reaction)

GM Level (g/kg)	Valid results	Number of positive partitions per reaction		
		0	1 or 2	≥ 3
0.45 g/kg	63	0	0	63

Table 3S: Absolute LOD of the MON810 specific dPCR module. Results of the analysis of sample levels in copies per reaction (cp/rxn)

GM Level (cp/rxn)	Valid results	Number of positive partitions per reaction		
		0	1 or 2	≥ 3
10	64	0	1	63
5	64	0	17	47
1	64	33	27	4

Table 4S: Absolute LOD of the hmg specific dPCR module. Results of the analysis of sample levels in copies per reaction (cp/rxn)

GM Level (cp/rxn)	Valid results	Number of positive partitions per reaction		
		0	1 or 2	≥ 3
10	64	0	2	62
5	64	1	16	47
1	64	27	31	6

Robustness

Table 5S: Detailed results of robustness tests for the 16 conditions and the change with respect to the optimised reaction conditions. (unch.= unchanged with respect to the optimised reaction conditions), the material with 1 g/kg MON810 was analysed

Condition	1	2	3	4	5	6	7	8	9	10	11	12	13	14	15	16
Ramp rate change (°C/s)	unch.	unch.	unch.	unch.	+ 0.5	+ 0.5	+ 0.5	+ 0.5	+ 0.5	+ 0.5	+ 0.5	+ 0.5	unch.	unch.	unch.	unch.
Annealing temp. change (°C)	unch.	unch.	unch.	unch.	unch.	unch.	unch.	unch.	+ 1	+ 1	+ 1	+ 1	+ 1	+ 1	+ 1	+ 1
Oligonucleotide concentration change	unch.	-10 %	-10 %	unch.	unch.	-10 %	-10 %	unch.	unch.	-10 %	-10 %	unch.	unch.	-10 %	-10 %	unch.
Master Mix concentration change	unch.	-10 %	unch.	- 10%	unch.	-10 %	unch.	- 10%	unch.	-10 %	unch.	- 10%	unch.	-10 %	unch.	- 10%
Mean (g/kg)	1.00	1.09	1.17	1.01	1.06	1.03	0.97	1.06	1.06	1.05	1.09	1.21	1.20	1.09	1.12	1.22
St. Dev. (g/kg)	0.18	0.15	0.32	0.09	0.14	0.18	0.19	0.16	0.24	0.17	0.25	0.33	0.14	0.23	0.29	0.19
Relative bias (%)	0.5%	9.3%	16.7%	0.9%	6.5%	2.8%	-2.8%	5.6%	5.6%	5.1%	9.3%	20.8%	20.4%	8.8%	11.6%	21.8%
RSD_r (%)	17.8%	13.5%	27.3%	9.3%	13.2%	17.7%	19.3%	15.6%	22.5%	16.6%	23.1%	27.3%	11.6%	20.7%	25.8%	15.6%

Section C: International collaborative trial

List of participating laboratories

- Bavarian Health and Food Safety Authority (LGL) (Oberschleissheim, Germany),
- Chemisches und Veterinäruntersuchungsamt Freiburg (Freiburg, Germany),
- Federal Food Safety and Veterinary Office FSVO (Bern, Switzerland),
- Istituto Superiore di Sanità (Roma, Italy),
- Istituto Zooprofilattico Sperimentale del Lazio e della Toscana "M. Aleandri" (Roma, Italy),
- Laboratorio Arbitral Agroalimentario (LAA) (Madrid, Spain),
- Landesamt für Verbraucherschutz Sachsen-Anhalt (Halle, Germany),
- LAVES - Niedersächsisches Landesamt für Verbraucherschutz und Lebensmittelsicherheit (Braunschweig, Germany),
- LGC (Teddington, United Kingdom),
- National Institute of Biology (Ljubljana, Slovenia),
- Office of Consumer Protection and Food Safety (BVL) (Berlin, Germany),
- Plant Health Laboratory ANSES (Angers CEDEX, France),
- Sciensano (Brussels, Belgium),
- Wageningen Food Safety Research (Wageningen, Netherlands).

Table 6S: List of equipment used by laboratories

Step	Equipment	No. of labs
Droplet generation	Biorad Droplet Generator	13
	Biorad AutoDG	1
PCR amplification	Biorad C1000 Touch Thermal Cycler	9
	Gene Amp PCR System 9700	3
	Bio-Rad T100 thermal cycler	2
Droplet Reader	BioRad QX200	11
	BioRad QX100	3
Droplet Reader software	QuantaSoft Version: 1.7.4.0917	14

List of deviations from the protocol:

- Due to concurrent engagements, one participant was allowed to report data after the stipulated deadline.
- One laboratory repeated the analysis of two samples due to issues during droplet generation resulted in low droplet counts.
- In one case, the two plates were read by two instruments due to an issue with the first instrument. No effect on the results was reported.
- One laboratory removed one value for technical reasons.
- One laboratory repeated the analysis of samples by simplex ddPCR from one plate because a number of wells showed low accepted droplet counts (< 10.000) and the analysis plot revealed an alternate atypical pattern. The repeated run showed expected plot patterns and satisfactory accepted droplet counts.
- One laboratory excluded two wells for the reference system in simplex ddPCR in one plate because of low accepted droplet counts.
- One laboratory reported technical problems and submitted results for two instead of four replicates per each concentration level for the simplex ddPCR format.

Table 7S: Results of duplex dPCR measurements submitted by participating laboratories. Detailed results (in % m/m) per each sample distributed containing relative mass fractions of MON810 from 0.1 % to 2 %

	GM mass fraction															
	0.1 %				0.5 %				0.9 %				2 %			
Laboratory	sample 1	sample 2	sample 3	sample 4	sample 1	sample 2	sample 3	sample 4	sample 1	sample 2	sample 3	sample 4	sample 1	sample 2	sample 3	sample 4
1	0.11	0.10	0.08	0.13	0.65	0.58	0.62	0.53	0.92	0.94	0.85	0.92	2.10	2.17	2.26	2.46
2	0.12	0.12	0.10	0.13	0.59	0.58	0.55	0.56	0.92	0.94	1.06	1.03	2.10	1.98	2.10	2.10
3	0.14	0.11	0.14	0.11	0.64	0.54	0.55	0.56	0.99	0.97	0.88	1.03	2.03	2.15	2.08	2.10
4	0.08	0.12	0.07	0.09	0.54	0.64	0.46	0.55	0.82	0.86	0.89	0.88	1.96	2.14	2.01	2.03
5	0.09	0.10	0.10	-	0.59	0.51	0.57	0.50	1.00	0.88	0.97	0.86	2.00	2.01	2.00	2.14
6	0.08	0.09	0.12	0.10	0.48	0.59	0.54	0.50	0.96	0.94	0.94	0.90	2.01	2.01	2.10	2.07
7	0.12	0.11	0.12	0.09	0.61	0.58	0.53	0.58	0.95	0.89	0.90	0.91	2.18	2.07	2.10	2.01
8	0.09	0.07	0.11	0.12	0.54	0.61	0.59	0.49	0.96	0.96	0.84	0.94	1.92	2.11	2.21	1.81
9	0.11	0.12	0.09	0.11	0.53	0.58	0.58	0.57	1.06	0.96	0.95	0.96	2.24	2.13	2.06	2.10
10	0.10	0.08	-	-	0.58	0.56	0.54	0.57	0.94	0.82	0.96	1.00	2.12	2.01	1.91	-
11	0.09	0.09	0.10	0.12	0.50	0.54	0.53	0.54	0.88	0.91	0.97	0.97	1.99	1.88	2.17	2.03
12	0.09	0.10	0.13	0.09	0.56	0.48	0.56	0.50	0.86	0.92	0.88	0.97	2.18	2.15	2.06	2.15
13	0.09	0.10	0.11	0.12	0.56	0.61	0.54	0.54	0.97	1.01	0.95	0.94	2.14	2.19	2.11	2.13
14	0.11	0.11	0.12	0.09	0.58	0.58	0.59	0.48	0.86	0.97	0.83	0.93	2.06	2.08	2.19	2.14

Table 8S: Results of duplex dPCR measurements submitted by participating laboratories. Detailed results (in % m/m) per each sample distributed with the 2 % relative mass fractions of MON810 material distributed as powder and analysed including the DNA extraction step (2 % ext) and 10 % relative mass fractions of MON810 material with different dilutions

	GM mass fraction															
	2 % ext				10 %				10 % dil 1:10				10 % dil 1:100			
Laboratory	sample 1	sample 2	sample 3	sample 4	sample 1	sample 2	sample 3	sample 4	sample 1	sample 2	sample 3	sample 4	sample 1	sample 2	sample 3	sample 4
1	2.03	2.42	2.11	2.08	9.92	10.13	10.24	9.82	10.28	9.72	10.83	10.56	7.36	9.58	9.44	8.75
2	2.15	1.94	2.05	2.00	10.44	10.51	10.05	10.24	10.33	10.32	9.89	10.24	7.68	12.19	11.34	11.31
3	2.17	1.78	2.07	2.19	10.03	10.32	10.15	10.01	11.11	9.58	10.42	10.14	15.83	11.53	12.36	8.89
4	1.91	1.91	2.00	1.92	9.61	9.74	9.76	9.76	10.83	9.04	9.45	9.35	12.26	7.71	11.05	10.13
5	2.25	1.90	1.89	1.99	10.32	10.29	9.85	10.24	9.86	10.14	10.00	10.97	9.31	8.89	9.58	12.36
6	1.85	2.01	1.81	1.83	10.22	9.97	10.29	10.24	11.11	9.86	10.56	9.06	10.83	11.67	11.67	10.69
7	1.78	1.86	2.32	1.83	10.15	10.18	10.26	9.93	10.97	10.56	9.86	9.58	10.56	11.11	8.33	11.39
8	2.24	2.28	2.00	2.06	9.94	10.15	9.94	10.15	10.42	10.42	10.00	9.44	7.08	10.83	9.31	9.17
9	2.06	2.16	2.04	2.06	9.83	9.70	10.50	10.23	10.40	9.92	8.89	11.03	8.40	9.61	7.39	10.19
10	2.01	2.04	2.16	1.79	10.05	9.97	9.77	10.19	10.87	10.98	8.80	9.90	8.94	9.58	7.83	7.30
11	2.11	1.92	2.24	2.14	10.36	9.93	10.01	10.18	10.83	10.56	10.28	8.85	10.14	11.39	10.14	6.94
12	1.75	1.94	1.97	1.94	10.39	10.11	10.11	10.03	9.44	-	10.10	9.21	9.86	12.36	9.31	10.69
13	1.94	2.00	1.96	1.85	10.49	10.24	10.26	10.21	9.14	10.82	10.35	10.42	10.00	11.94	11.94	8.33
14	2.07	1.88	2.04	2.04	9.90	9.99	10.33	10.17	10.00	10.14	9.72	9.86	9.86	11.94	8.47	9.31

Table 9S: Results of simplex dPCR measurements submitted by participating laboratories. Detailed results (in % m/m) per each sample distributed containing relative mass fractions of MON810 from 0.1 % to 2 %

	GM mass fraction															
	0.1 %				0.5 %				0.9 %				2 %			
Laboratory	sample 1	sample 2	sample 3	sample 4	sample 1	sample 2	sample 3	sample 4	sample 1	sample 2	sample 3	sample 4	sample 1	sample 2	sample 3	sample 4
1	0.08	0.09	0.13	0.08	0.57	0.58	0.58	0.53	0.97	1.01	0.98	0.82	2.16	1.94	2.14	1.94
2	0.09	0.11	0.10	0.15	0.58	0.55	0.57	0.58	0.94	0.99	0.88	0.94	2.18	2.05	1.97	2.09
3	0.11	0.09	0.10	0.10	0.61	0.61	0.58	0.50	0.94	0.85	0.98	0.95	2.00	2.14	2.21	2.02
4	0.13	0.10	0.08	0.11	0.55	0.59	0.56	0.48	0.86	0.77	0.80	0.93	1.97	1.99	1.96	1.82
5	0.11	0.10	0.16	0.14	0.56	0.55	0.59	0.58	0.94	0.95	1.00	1.01	2.14	2.14	2.12	2.17
6	0.11	0.13	0.13	0.10	0.61	0.57	0.71	0.53	0.97	0.87	0.92	0.99	2.02	2.06	2.04	2.02
7	0.12	0.13	0.12	0.09	0.54	0.63	0.54	0.62	1.11	0.93	0.98	1.10	2.10	2.00	2.04	2.18
8	0.10	0.09	0.12	0.10	0.59	0.50	0.59	0.49	1.00	0.96	0.93	0.85	1.84	2.02	1.99	2.04
9	0.11	0.10	0.11	0.16	0.55	0.64	0.59	0.54	0.90	0.99	0.87	1.04	1.96	2.31	2.17	2.05
10	0.09	0.13	-	-	0.54	0.57	-	-	0.82	0.99	-	-	1.86	1.85	-	-
11	0.09	0.10	0.12	0.12	0.63	0.53	0.62	0.64	1.02	1.02	0.98	0.92	2.28	2.03	2.12	2.10
12	0.12	0.08	0.07	0.15	0.52	0.61	0.56	0.69	0.94	0.89	1.07	0.97	1.99	2.32	2.14	1.96
13	0.10	0.11	0.11	0.12	0.56	0.57	0.61	0.56	0.95	0.87	0.88	0.97	1.97	2.11	2.16	2.11
14	0.11	0.13	0.12	0.11	0.48	0.56	0.52	0.67	0.96	0.77	0.97	0.83	2.32	1.92	1.74	1.80

Table 10S: Results of simplex dPCR measurements submitted by participating laboratories. Detailed results (in % m/m) per each sample distributed containing a relative MON810 mass fraction of 10 % with different dilutions

	GM mass fraction											
	10 %				10 % dil 1:10				10 % dil 1:100			
Laboratory	sample 1	sample 2	sample 3	sample 4	sample 1	sample 2	sample 3	sample 4	sample 1	sample 2	sample 3	sample 4
1	10.13	9.69	9.43	9.70	9.08	9.23	9.40	10.18	9.82	8.65	8.71	8.49
2	10.29	10.38	10.75	10.08	10.73	10.38	10.67	10.57	6.90	9.54	8.88	7.41
3	9.63	9.79	10.47	10.28	10.52	10.85	10.31	10.02	12.66	9.21	10.64	8.38
4	9.97	9.61	9.27	9.81	9.47	9.32	9.03	8.69	9.89	9.47	8.10	9.50
5	10.58	10.34	10.20	10.38	9.16	10.00	10.80	10.38	10.84	10.54	8.63	12.22
6	9.75	10.43	11.03	10.29	11.61	10.18	9.41	10.66	9.94	8.63	10.96	9.09
7	10.24	10.03	9.95	9.95	10.96	10.74	9.72	10.34	9.44	8.02	10.71	10.22
8	9.30	10.50	10.59	10.08	9.45	10.20	9.34	9.87	8.98	10.57	9.71	12.98
9	9.83	10.28	10.58	9.66	9.69	10.79	10.18	9.70	9.63	11.18	10.56	11.23
10	9.81	10.54	-	-	10.25	8.97	-	-	8.24	11.36	-	-
11	10.25	10.42	10.30	9.59	10.78	10.43	11.24	10.81	10.18	10.30	12.94	10.76
12	10.63	10.25	9.37	10.13	10.66	9.65	9.38	10.55	9.35	10.36	13.97	10.30
13	10.79	10.57	10.02	10.12	10.65	10.24	11.69	10.00	8.07	8.74	13.16	8.30
14	11.28	9.86	9.35	11.21	9.59	8.76	12.31	9.19	9.61	12.46	12.43	12.92
